# B7-H3 expression in colorectal cancer: associations with clinicopathological parameters and patient outcome

**DOI:** 10.1186/1471-2407-14-602

**Published:** 2014-08-20

**Authors:** Vibeke A Ingebrigtsen, Kjetil Boye, Jahn M Nesland, Arild Nesbakken, Kjersti Flatmark, Øystein Fodstad

**Affiliations:** Department of Tumor Biology, Norwegian Radium Hospital, Oslo University Hospital, PO Box 4950, Nydalen, N-0424 Oslo, Norway; Institute for Clinical Medicine, Faculty of Medicine, University of Oslo, PO Box 1171, Blindern, N-0318 Oslo, Norway; Department of Oncology, Norwegian Radium Hospital, Oslo University Hospital, PO Box 4950, Nydalen, N-0424 Oslo, Norway; Department of Pathology, Norwegian Radium Hospital, Oslo University Hospital, PO Box 4950, Nydalen, N-0424 Oslo, Norway; Department of Gastroenterological Surgery, Norwegian Radium Hospital, Oslo University Hospital, PO Box 4950, Nydalen, N-0424 Oslo, Norway; Department of Gastrointestinal Surgery, Oslo University Hospital – Aker, PO Box 4950, Nydalen, N-0424 Oslo, Norway

**Keywords:** Colorectal cancer, Nuclear B7-H3, Prognostic biomarker, Tissue microarray

## Abstract

**Background:**

We have previously reported overexpression of the immunoregulatory protein B7-H3 in colorectal cancer and that nuclear expression predicted poor outcome in colon cancer patients. The present study was performed to examine the prognostic role of B7-H3 in an independent colorectal cancer cohort.

**Methods:**

Using tissue microarrays from 731 colorectal cancer patients, tumour B7-H3 expression was assessed by immunohistochemistry. Associations with clinicopathological parameters and patient outcome were investigated.

**Results:**

Nuclear expression of B7-H3 in cancer cells was present in 27% of the samples in the total study cohort, while cytoplasmic/membrane and stromal expression was seen in 86% and 77% of the samples, respectively. Nuclear B7-H3 had no prognostic relevance in the complete outcome cohort, neither in colon cancer patients. However, nuclear B7-H3 was significantly associated with reduced recurrence-free survival in TNM stage I colorectal cancer patients.

**Conclusions:**

Overexpression of B7-H3 in colorectal cancer was confirmed, but in contrast to previous results, nuclear B7-H3 was not a strong prognostic biomarker in this cohort. The discrepancy might be related to the use of single-core tissue microarrays for detection of the heterogeneously expressed B7-H3, and the role of B7-H3 in colorectal cancer still needs further examination.

**Electronic supplementary material:**

The online version of this article (doi:10.1186/1471-2407-14-602) contains supplementary material, which is available to authorized users.

## Background

Colorectal cancer (CRC) is a major cause of cancer-related morbidity and mortality worldwide. Yearly more than one million new cases of CRC are diagnosed and about 600 000 patients succumb to the disease, and this makes it the fourth most common cause of cancer deaths [1]. Surgery is the cornerstone treatment for CRC, and while node-positive (TNM III) patients generally profit from adjuvant chemotherapy, no clear survival benefit has been shown for patients with stage I and II disease [2, 3]. However, tumours within the same stage are biologically and molecularly heterogeneous, and a significant proportion of stage II patients develop metastatic disease despite apparently curative surgery [4, 5]. Thus, reliable prognostic biomarkers are needed to complement the TNM system in identifying patients with increased risk of disease recurrence. Great effort has been made to find such markers. Microsatellite instability (MSI) [6–8] and loss of the DCC (Deleted in Colon Cancer) gene [9] are promising candidates, but there is not yet sufficient evidence to apply them in clinical treatment decisions.

B7-H3, an immunoregulatory protein in the B7 family of T cell co-regulatory molecules [10, 11], is overexpressed in several different cancer forms including CRC [12–20]. The precise role of B7-H3 in tumour immunity is still ambiguous, as both T cell co-stimulatory and co-inhibitory effects have been demonstrated [21, 22]. Our group has previously demonstrated that B7-H3 silencing conferred reduced metastatic capacity and increased chemosensitivity in both *in vitro* and *in vivo* models [23–25], implying that B7-H3 is involved in cancer progression also via non-immunological mechanisms. This is supported by the work of Wang *et al*. and Zhao *et al.*, which showed that knock down of B7-H3 leads to reduced cell migration and invasion [26, 27], as well as increased gemcitabine sensitivity [28].

We have previously evaluated B7-H3 expression in nearly 300 CRC whole tissue sections (WTSs) and found that it was expressed in the tumour cell nucleus in about 30% of the tumours [19]. Interestingly, nuclear localisation of B7-H3 strongly predicted poor outcome in colon cancer, suggesting that nuclear B7-H3 might be considered a new biomarker that could facilitate colon cancer treatment decisions, pending validation of the results in other CRC cohorts. The aim of the present work was therefore to evaluate associations between tumour B7-H3 expression and clinicopathological parameters and patient outcome in an independent cohort of CRC patients.

## Methods

### Patient cohort and tissue microarray

Formalin-fixed, paraffin-embedded tumour tissue was prospectively collected for all patients (n = 939) undergoing primary surgery for CRC at Oslo University Hospital-Aker between 1993 and 2003. Registration has been controlled against the Norwegian Cancer Registry. Representative tumour tissue was collected for TMA construction with 1.0 mm diameter cores. The study was approved by the Regional Committee for Medical and Health Research Ethics (REK1.2005.1629).

Two hundred and eight patients were excluded for the following reasons: not representative or no tumour tissue (183), technically unsuccessful staining (12), unknown disease stage (12), and not primary tumour (1). The total study population thus included 731 patients with histologically verified primary CRC. The outcome study population included 562 patients in TNM stages I-III who had undergone curative surgery, with 402 (72%) of the tumours localised in the colon and 156 (28%) in the rectum. Mean patient age at the time of surgery in both the total and the outcome study cohort was 73 years (range: 30–94 years). Colon cancer patients younger than 76 years and all rectal cancer patients entered a 5-year follow-up program. Patients who were not enrolled in systematic follow-up (n = 353) would be admitted to the hospital for symptoms of relapse, and it is assumed that nearly all relapses would be identified and registered. Further details have previously been described [29].

Recurrence-free survival was measured from the date of surgery until diagnosis of locoregional recurrence or distant metastases within the first 5 years after surgery. Of the 562 patients in the outcome study cohort, 115 patients (20%) were diagnosed with locoregional recurrence or distant metastasis during this period. Patients without locoregional recurrence or distant metastases were censored at time of death. Overall survival was measured from date of surgery until death. Survival data were obtained from the National Registry of Norway. During the total follow-up period 332 patients passed away. Median follow-up of patients still alive was 9.7 years (range: 5.2 - 17.3 years).

### Immunohistochemistry

Representative cylindrical tissue cores from formalin-fixed and paraffin-embedded colorectal tumours were selected by an experienced colorectal pathologist and collected in a tissue microarray (TMA). The TMA sections were immunostained using the Dako EnVision FLEX + detection system (Dako, Glostrup, Denmark). Dako PT link was used for deparaffinisation and heat-induced epitope retrieval. Sections were preheated in Dako EnVision FLEX + Target Retrieval Solution, High pH, and rinsed in Dako wash buffer according to the manufacturer’s instructions. Endogenous peroxidase activity was blocked with EnVision FLEX Peroxidase-Blocking Reagent, before incubation with mouse monoclonal anti-human B7-H3 antibody (Clone MIH42, LifeSpan BioSciences, Seattle, WA), at 1:300 for 30 minutes at room temperature. The primary antibody signal was amplified by EnVision FLEX + Mouse (LINKER) before incubation with EnVision FLEX/HRP Detection Reagent for 30 minutes. Finally, the sections were incubated with diaminobenzene before they were counterstained with hematoxylin, rinsed and mounted in Cytoseal XYL (Thermo Scientific, Waltham, MA). Negative controls included replacement of the primary antibody with normal polyclonal mouse IgG of the same subclass and concentration, and incubations of sections with mouse monoclonal anti-B7-H3 antibody pre-absorbed with 10 μg/ml recombinant human B7-H3 (R&D Systems, Minneapolis, MN). Positive controls (sections from formalin-fixed and paraffin-embedded colorectal tumour tissue known to express high amounts of B7-H3) were included in all series.

The immunostained TMAs were scored microscopically by the study pathologist (J.M.N), without knowledge of patient outcome or corresponding clinicopathological parameters. Nuclear staining in tumour cells was recorded as an individual variable. As membrane positive tumour cells typically displayed cytoplasmic staining as well, cytoplasmic and membrane staining were recorded as one variable, as were staining of tumour-associated fibroblasts and endothelial cells. Nuclear and stromal staining was scored as negative (no staining) or positive (nuclear/stromal staining present). Cytoplasmic and membrane staining was semi-quantitatively estimated, and graded as 0 (no staining), 1 (less than 10% positive cells), 2 (10-49% positive cells) or 3 (more than 50% positive cells). For the statistical analyses the cytoplasmic/membrane B7-H3 scores were divided into negative (no staining) and positive staining (cytoplasmic/membrane staining present). The rationale for choosing this cut-off is that the TMA cores only comprise a small section of the tumour, and even a few positive cells in the core indicate a B7-H3 positive tumour.

### Statistical analysis

Associations between B7-H3 expression and clinicopathological parameters were analysed using two-tailed Fisher’s exact test or linear-by-linear association chi-square test. Survival analysis was performed according to the Kaplan-Meier method, and survival was compared using the log rank test. The SPSS version 18.0 software (SPSS, Chicago, IL) was used for statistical calculations. P-values below 0.05 were considered statistically significant. Five patients had duplicate cores and of these, three had contradictory nuclear B7-H3 score in the two cores. Scoring these three patients as nuclear positive or negative or excluding them from statistical analysis did not significantly influence results, and in the presented results, nuclear score was set to positive for these cases. For 5 patients in the total study cohort and 4 patients in the outcome study cohort tumour localisation was not known, and these were excluded from statistical analyses involving tumour localisation.

## Results

### Patient characteristics

Clinical and pathological features for the total and the outcome study population are summarised in Table 1. The total study cohort included 389 (53%) females and 342 (47%) males. The majority of the patients was in TNM stage II (307/42% in the total study cohort and 287/51% in the outcome study cohort), had pT3 tumours (73% in both cohorts) and no lymph node metastases (63 and 69%, respectively). Most tumours were intermediately differentiated (78% in both cohorts). In both cohorts 103 patients had TNM stage I disease.

**Table 1:** **Baseline clinicopathological data and tumour expression of B7-H3**

		Total study cohort		Outcome study cohort	
		Patients		Patients	
Parameter		Number	%	Number	%
**Gender**	Female	389	53	292	52
	Male	342	47	270	48
**TNM stage**	I	103	14	103	18
	II	307	42	287	51
	III	186	25	172	31
	IV	135	18	-	-
**pT**	1	27	4	26	5
	2	98	13	94	17
	3	534	73	410	73
	4	72	10	32	6
**pN**	0	455	63	389	69
	1	197	27	135	24
	2	74	10	37	7
	ND	5	-	1	-
**Differentiation**	Well	72	10	66	12
	Intermediate	549	78	426	78
	Poor	87	12	55	10
	ND	23	-	15	-
**Tumour localisation**	Colon	546	75	402	72
	Rectum	180	25	156	28
	Unknown	5	-	4	-
**Cytoplasmic/membrane B7-H3**	0	104	14	74	13
	1	79	11	44	8
	2	124	17	101	18
	3	424	58	343	61
**Nuclear B7-H3**	0	534	73	400	71
	1	197	27	162	29
**Total** ^**1**^ **B7-H3**	0	94	13	66	12
	1	637	87	496	88
**Stromal B7-H3**	0	171	23	121	22
	1	560	77	441	78

*Abbreviations*: *ND* not determined, *pN* pathological nodal stage, *pT* pathological tumour stage, *TNM* tumour node metastasis.

^1^Nuclear, and/or cytoplasmic/membrane staining.

### B7-H3 expression in primary colorectal carcinomas

**Figure 1:**
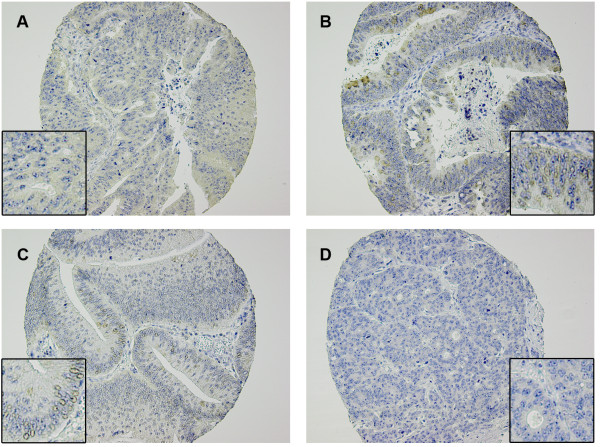
**Representative photomicrographs of colorectal cancer TMA specimens stained with anti-B7-H3 antibody.** Panel **A** shows predominantly cytoplasmic and stromal staining, panel **B** shows nuclear and cytoplasmic staining, panel **C** shows nuclear staining and panel **D** shows a B7-H3 negative tumour.

The immunohistochemical expression levels of B7-H3 are shown in Table 1, and representative microscope images are shown in Figure 1. The majority of tumours displayed cytoplasmic/membrane staining (Figure 1A and B) (86% in the total study cohort), as well as stromal staining (Figure 1A) (77% in the total study cohort). Nuclear staining was seen in 27% of the evaluated samples in the total study cohort (Figure 1B and C).

### Associations between B7-H3 expression and clinicopathological parameters

No significant associations were found between cancer cell B7-H3 expression and clinicopathological parameters, as shown in Table 2. However, absence of stromal B7-H3 expression was associated with advanced TNM stage (p = 0.03) and the presence of lymph node metastases pN (p = 0.007). We did not observe any differences in B7-H3 expression in colon versus rectal cancer.

**Table 2:** **Associations between B7-H3 expression and clinicopathological parameters – total study cohort**

Parameter		Patients number (%)	Cytoplasmic/membrane B7-H3	Nuclear B7-H3	Total ^1^ B7-H3	Stromal B7-H3
**Gender**	Male	342 (47)	298 (87)	92 (27)	303 (89)	269 (79)
	Female	389 (53)	329 (85)	105 (27)	334 (86)	291 (75)
	*p-value*		*0.34*	*1.0*	*0.32*	*0.26*
**TNM**	I	103 (14)	88 (85)	33 (32)	89 (86)	79 (77)
	II	307 (42)	268 (87)	78 (25)	272 (89)	250 (81)
	III	186 (25)	161 (87)	58 (31)	165 (89)	137 (74)
	IV	135 (18)	110 (81)	28 (21)	111 (82)	94 (70)
	*p-value*		*0.29*	*0.22*	*0.27*	*0.03*
**pT**	1	27 (4)	20 (74)	9 (33)	22 (81)	20 (74)
	2	98 (13)	86 (88)	32 (33)	86 (88)	73 (74)
	3	534 (73)	463 (87)	135 (25)	471 (88)	419 (78)
	4	72 (10)	58 (81)	21 (29)	58 (81)	48 (67)
	*p-value*		*0.93*	*0.34*	*0.72*	*0.62*
**pN**	0	455 (63)	393 (86)	119 (26)	398 (87)	363 (80)
	1	197 (27)	169 (86)	61 (31)	173 (88)	147 (75)
	2	74 (10)	62 (84)	16 (22)	63 (85)	49 (66)
	*p-value*		*0.58*	*1.0*	*0.74*	*0.007*
**Differentiation**	Well	72 (10)	62 (86)	25 (35)	63 (88)	54 (75)
	Intermediate	549 (78)	469 (85)	144 (26)	477 (87)	423 (77)
	Poor	87 (12)	74 (85)	22 (25)	75 (86)	67 (77)
	*p-value*		*0.91*	*0.21*	*0.82*	*0.85*
**Tumour localisation** ^**2**^	Colon	546 (75)	473 (87)	150 (27)	481 (88)	417 (76)
	Rectum	180 (25)	150 (83)	46 (26)	152 (84)	141 (78)
	*p-value*		*0.34*	*0.7*	*0.2*	*0.61*

The table shows the number/percent of patients in each subcategory, the number/percent of B7-H3 positive patients in each subcategory as well as the *p-value* resulting from statistical analysis of associations between B7-H3 expression and each parameter.

^1^Nuclear and/or cytoplasmic/membrane staining.

^2^Five patients had unknown tumour localisation, these were excluded from the statistical analyses involving tumour localisation.

### Associations between clinicopathological parameters and outcome

The prognostic significance of clinicopathological parameters was investigated by univariate analysis (Table 3). Advanced TNM stage, T classification and nodal status were significantly associated with the development of locoregional recurrence or distant metastases within the first 5 years after surgery. There was a tendency towards increased recurrence-free survival for colon cancer patients versus rectal cancer patients, but it did not reach statistical significance (p = 0.13). In accordance with the results for recurrence-free survival, TNM stage, pT and pN were also significantly associated with overall survival.

**Table 3:** **Associations between survival and clinicopathological parameters and B7-H3 expression**

	Univariate analysis (P-value, log rank test)	
	Recurrence-free survival	Overall survival
Gender	0.26	0.34
TNM stage	<0.001	<0.001
pT	<0.001	<0.001
pN	<0.001	<0.001
Differentiation	0.58	0.38
Tumour localisation^1^	0.13	0.33
Cytoplasmic/Membrane B7-H3	0.25	0.82
Nuclear B7-H3	0.62	0.50
Total^2^ B7-H3	0.24	0.90
Stromal B7-H3	0.43	0.84

^1^Five patients had unknown tumour localisation, these were excluded from the statistical analyses involving tumour localisation.

^2^Nuclear and/or cytoplasmic/membrane staining.

### Associations between B7-H3 expression and patient outcome

**Figure 2:**
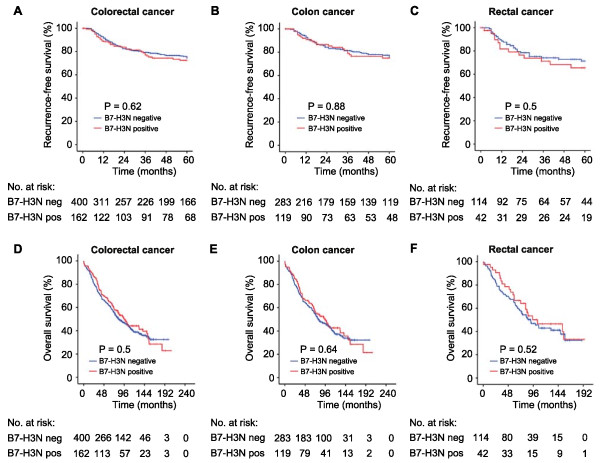
**Kaplan-Meier survival plots presenting recurrence-free survival (upper row) and overall survival (lower row) based on nuclear expression of B7-H3 (B7-H3N) in tumour specimens from colorectal cancer (A, D), colon cancer (B, E) and rectal cancer patients (C, F).**

**Figure 3:**
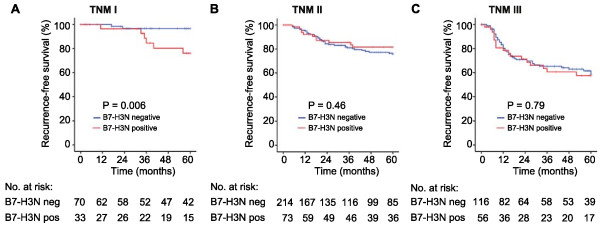
**Kaplan-Meier survival plots presenting recurrence-free survival based on nuclear expression of B7-H3 (B7-H3N) in tumour specimens from colorectal cancer patients in TNM stage I (A), TNM stage II (B) and TNM stage III (C).**

Univariate analysis did not display significant associations between B7-H3 expression and patient outcome in CRC patients (Table 3). In contrast to what was found in the previous WTS study nuclear B7-H3 had no prognostic relevance in the complete outcome cohort (p = 0.62 for recurrence-free survival and 0.5 for overall survival), neither when analysing colon (p = 0.88 for recurrence-free survival and 0.64 for overall survival) and rectal patients (p = 0.5 and 0.52, respectively) separately (Figure 2). However, in TNM stage I patients there was a strong association between the presence of nuclear B7-H3 expression and reduced recurrence-free survival (p = 0.006, Figure 3), but not with overall survival (p = 0.57, data not shown).

### Simulated tissue microarray cores from whole tissue sections

**Figure 4:**
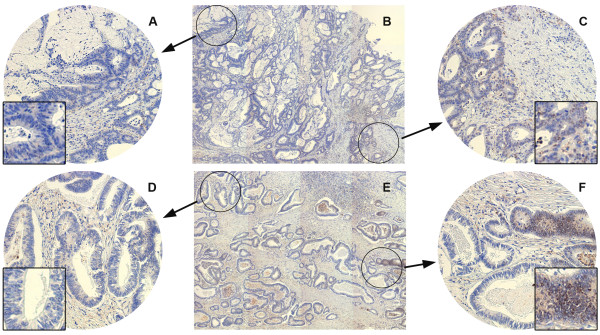
**Immunohistochemical staining of colorectal cancer WTS specimens with anti-B7-H3 antibody.** Panels **A, C, D** and **F** show simulated TMA cores from distinct segments of the whole tissue sections from patient 16 **(B)** and patient 30 **(E)**. The left panels **(A, D)** show nuclear negative cores, and the right panels **(C, F)** show nuclear positive cores.

As the previously observed prognostic significance of nuclear B7-H3 staining could not be confirmed in the present TMA cohort, we wanted to assess whether the use of TMAs instead of WTSs to evaluate tumour B7-H3 expression might have affected the results, and we looked in more detail at the nuclear expression pattern in a few of the old WTS sections. Figure 4 shows B7-H3 immunohistochemical staining in two simulated TMA cores from distinct segments of the WTSs from patient 16 and 30 in the WTS cohort. As is evident in the images, there is considerable heterogeneity of nuclear B7-H3 expression in the depicted CRC tumour samples, and the selection of two morphologically representative cores from each tumour resulted in opposite nuclear B7-H3 score. This example clearly demonstrates the inherent problem in obtaining TMA cores representative for the entire tumour.

## Discussion

We have previously reported that nuclear B7-H3 protein expression was strongly and independently associated with poor prognosis in colon cancer patients [19]. In the present work we examined immunohistochemical expression of B7-H3 in TMAs prepared from a large panel of colorectal carcinomas, and evaluated the associations between B7-H3 expression, clinicopathological parameters and patient outcome. The main objective was to investigate whether the results from our previous study could be validated in an independent CRC cohort.

In the present work, nuclear B7-H3 was significantly associated with reduced recurrence-free survival in TNM I patients only, contrasting the previous study in which nuclear B7-H3 was significantly and independently associated with reduced metastasis-free and overall survival in colon cancer patients. The inconsistency between present and previous results on the prognostic impact of nuclear B7-H3 may have several possible explanations. Differences between the two examined CRC patient cohorts could exist, but both were prospectively established from patients undergoing surgery for primary CRC within the same time period, and with no substantial differences in cohort composition (Additional file 1: Table S1). There was, however, a critical methodological difference between the two studies, since TMAs were used in the present work compared to WTSs in the previous one. The use of single, small cores for TMA construction may not be representative of large tumours in the colorectum, in particular when the protein of interest exhibits significant expression heterogeneity [30, 31]. Using the simulated TMAs as examples, we show that because of heterogeneity of nuclear B7-H3 expression in the CRC tumour samples, conclusions regarding nuclear B7-H3 status partially depend on where the core was taken. Similar results have been demonstrated in a study comparing WTSs and TMAs in clear cell renal cell carcinoma using simulated TMAs. The number of cores required to adequately represent WTS quantification was shown to be biomarker specific, and for B7-H3 2–3 cores appeared necessary [32]. Thus, although the present cohort represents a large, well-characterized population-based collection of CRC specimens, heterogeneous expression of the protein of interest rendered the single-core TMA approach questionable in this setting, and this represents a limitation for the interpretation of the results.

In both the previous and the present study nuclear B7-H3 staining was detected in nearly one third of the evaluated tumour samples. While nuclear expression of B7-H3 in tumour cells so far has been reported by our group only, several authors have described the prognostic impact of tumour B7-H3 expression in various other cancer forms. The results are highly variable and even conflicting: Most reports indicate that high tumour B7-H3 level is associated with advanced disease and/or poor outcome [13, 14, 16, 17, 20, 33, 34]. Some investigators report no associations between tumour B7-H3 and prognosis [35, 36], and some that high tumour B7-H3 expression is associated with improved outcome [12, 15]. Taken together, this indicates that the role of B7-H3 as a prognostic marker in cancer in general is undetermined. Though the inconsistent findings might be due to methodological issues it could also be that B7-H3 has different prognostic and functional roles in different cancer forms.

Nuclear B7-H3 was associated with poor outcome in TNM I patients in this cohort, and the strongest association between nuclear B7-H3 and reduced survival in the previous cohort was seen in TNM II patients. This might hypothetically point to nuclear B7-H3 as a possible promoter of progression and metastasis in early stages of CRC. It is well known that cancer associated proteins can have diverse roles in the different stages of cancer progression [37, 38]. Additionally, differences regarding the expression level and distribution of a protein between cancer cells and stromal cells in a tumour may reflect shifts in biological effects. Hence, that absent stromal B7-H3 was associated with advanced disease stage at diagnosis might be of some interest. T cell co-stimulatory effects of B7-H3 have been demonstrated, and theoretically tumours lacking stromal B7-H3 could escape anti-tumour immunity which would otherwise impede disease progression [39, 40]. On the other hand, B7-H3 suppression of anti-tumour immunity has also been reported [18, 41, 42], leaving the immunomodulatory role of B7-H3 in this setting unclear. Another hypothesis could be that stromal B7-H3 restrains disease progression through so far unknown non-immunological mechanisms. Despite uncertainty regarding the clinical implications of nuclear and stromal B7-H3 in CRC, our findings may be important to consider in future studies.

## Conclusions

Nuclear B7-H3 was not a strong prognostic biomarker in CRC in the present study, contrasting previous findings by our group. Because of heterogeneous expression of nuclear B7-H3, the use of single-core TMAs instead of WTSs in the present study may have influenced the results. However, our results and the findings of other groups might indicate that hypothetically the biological role of B7-H3 may differ from one tumour type to another, may change during disease progression, and furthermore that the protein might attain different functions dependent on cell type, microenvironment and subcellular localisation. Together with data indicating that B7-H3 promotes cancer progression through modulation of important signaling pathways [24, 25] this calls for further investigation.

## Electronic supplementary material

Additional file 1: **Clinicopathological parameters and B7-H3 expression, WTS and TMA cohorts (outcome study cohorts).** (DOC 48 KB)

## Abbreviations

CRC: Colorectal cancer
DCC: Deleted in colon cancer
MSI: Microsatellite instability
TMA: Tissue microarray
TNM: Tumour node metastasis
WTS: Whole tissue section.
