# Supplementary material for: B7-H3 expression in colorectal cancer: associations with clinicopathological parameters and patient outcome
Source: BMC Cancer. 2014 Aug 20;14:602. doi: 10.1186/1471-2407-14-602 (PMC4148536; doi:10.1186/1471-2407-14-602)
Supplement: Supplementary file 1 — Additional file 1: Clinicopathological parameters and B7-H3 expression, WTS and TMA cohorts (outcome study cohorts). (DOC 48 KB) [file 12885_2013_4784_MOESM1_ESM.doc]

**Additional file 1. Clinicopathological parameters and B7-H3 expression, WTS and TMA cohorts (outcome study cohorts)**

|  |  | **Whole section cohort** | **TMA cohort** |
| --- | --- | --- | --- |
| **N** |  | 242 | 562 |
| **Age (years)** | Median (range) | 73 (35–98) | 73 (30–94) |
| **Gender** | Female | 110 (45%) | 292 (52%) |
|  | Male | 132 (55%) | 270 (48%) |
| **TNM stage** | I | 53 (22%) | 103 (18%) |
|  | II | 112 (46%) | 287 (51%) |
|  | III | 77 (32%) | 172 (31) |
| **pT** | 1 | 8 (3%) | 26 (5%) |
|  | 2 | 51 (21%) | 94 (17%) |
|  | 3 | 159 (66%) | 410 (73%) |
|  | 4 | 24 (10%) | 32 (6%) |
| **pN** | 0 | 165 (68%) | 389 (69%) |
|  | 1 | 51 (21%) | 135 (24%) |
|  | 2 | 26 (11%) | 37 (7%) |
|  | ND | - | 1 |
| **Differentiation** | Well | 7 (3%) | 66 (12%) |
|  | Intermediate | 208 (86%) | 426 (78%) |
|  | Poor | 27 (11%) | 55 (10%) |
|  | ND | - | 15 |
| **Tumour localisation** | Colon | 163 (67%) | 402 (72%) |
|  | Rectum | 79 (33%) | 156 (28%) |
|  | Unknown | - | 4 (1%) |
| **Outcome** | Metastasis/recurrence | 57 (24%) | 115 (20%) |
|  | Death | 116 (48%) | 332 (59%) |
| **Follow-up (years), patients still alive** | Median (range) | 9.0 (8.2-10.0) | 9.7 (5.2-17.3) |
| **Cytoplasmic/**  **membrane B7-H3** | 0 | 90/132 (38/56%)^1^ | 74 (13%) |
|  | 1-3 | 148/106 (62/44%)^1^ | 488 (87%) |
| **Nuclear B7-H3** | 0 | 165 (69%) | 400 (71%) |
|  | 1 | 73 (31%) | 162 (29%) |
| **Total B7-H3** | 0 | 68 (29%) | 66 (12%) |
|  | 1 | 170 (71%) | 496 (88%) |
| **Stromal B7-H3 (endothelial/fibroblast)** | 0 | 19/28 (8/12%)^2^ | 121 (22%) |
|  | 1 | 219/210 (92/88%)^2^ | 441 (78%) |

Abbreviations: ND = not determined; pN = pathological nodal stage; pT = pathological tumour stage; TNM stage = tumour node metastasis stage

^1^Cytoplasmic/membrane B7-H3 were recorded as separate variables in the WTS cohort and as one variable in the TMA cohort

^2^Endothelial/fibroblast B7-H3 were recorded as separate variables in the WTS cohort and as one variable in the TMA cohort
